# Doxorubicin Loaded Magnesium Oxide Nanoflakes as pH Dependent Carriers for Simultaneous Treatment of Cancer and Hypomagnesemia

**DOI:** 10.3390/nano9020208

**Published:** 2019-02-06

**Authors:** Tharindu A. Ranathunge, D.G.G.P. Karunaratne, R.M.G. Rajapakse, Davita L. Watkins

**Affiliations:** 1 Department of Chemistry, University of Peradeniya, Kandy 20400, Sri Lanka; garanath@go.olemiss.edu; 2 Department of Chemical and Processing Engineering, University of Peradeniya, Kandy 20400, Sri Lanka; dpkaru@eng.pdn.ac.lk; 3 Postgraduate Institute of Science, University of Peradeniya, Kandy 20400, Sri Lanka; 4 Department of Chemistry and Biochemistry, The University of Mississippi, University, MS 38677, USA

**Keywords:** MgO nanoparticles, doxorubicin, nanoflakes, drug delivery

## Abstract

Doxorubicin (DOX) is an anticancer drug commonly used in treating cancer; however, it has severe cytotoxicity effects. To overcome both the adverse effects of the drug and mineral deficiency (i.e., hypomagnesemia) experienced by cancer patients, we have developed magnesium oxide (MgO) nanoflakes as drug carriers and loaded them with DOX for use as a targeted drug delivery (TDD) system for potential application in cancer therapy. The synthesis employed herein affords pure, highly porous MgO nanoparticles that are void of the potentially harmful metal contaminants often discussed in the literature. Purposed for dual therapy, the nanoparticles exhibit an impressive 90% drug loading capacity with pH dependent drug releasing rates of 10% at pH 7.2, 50.5% at pH 5.0, and 90.2% at pH 3. Results indicate that therapy is achievable via slow diffusion where MgO nanoflakes degrade (i.e., dissolve) under acidic conditions releasing the drug and magnesium ions to the cancerous region. The TDD system therefore minimizes cytotoxicity to healthy cells while supplying magnesium ions to overcome hypomagnesemia.

## 1. Introduction

Cancer is one of the leading causes of death, accounting for an annual mortality rate of 163.5 per 100,000 men and women as per 2011–2015 death statistics [1]. Chemotherapy stands as one of the most important treatment strategies. However, chemotherapy is associated with severe drawbacks. Among those, toxicity to healthy cells is a major disadvantage as it leads to complications such as hair loss, nausea, fatigue, and increased risk of infection [2,3,4]. As such, development of non-toxic drug carriers capable of high drug encapsulation with minimal cargo loss during circulation, yet slow and steady release solely at the cancer cells is desirable.

Cancer cells have much higher metabolic rates than healthy normal cells, tending to consume more nutrients and oxygen [5]. An insufficient supply of oxygen to cancer cells causes acidosis producing lactic acid at the extracellular medium. In turn, the extracellular pH of cancer cells is in the range of 6.2–6.9, while those of intracellular medium are in the range of 7.12–7.65 [6]. Therefore, the extracellular medium of cancerous cells are slightly acidic and any non-toxic material that would slowly dissolve in such conditions would be useful as drug carriers.

Non-toxic inorganic nanoparticles, such as CaCO_3_ and MgO, which dissolve at slightly acidic conditions, fulfill these requirements and can be used as drug carriers for transporting anticancer drugs for targeted delivery (TD) and slow release (SR). Since these materials do not dissolve at the physiological pH of blood (7.2), drug release is less likely to occur during the transport process. Instead, it would happen slowly at the extracellular medium of cancerous cells. Such a strategy is useful in reducing cytotoxicity of the drugs to healthy cells. In addition, it helps to minimize the dosage requirement and wastage of the drug while enhancing its efficacy and bio-availability. Recently, we tested the hypothesis using model drug copper bis(8-hydroxyquinoline) in hydroxyapatite nanoparticles and found it to be a viable approach to TD [6]. We then developed vaterite forms of CaCO_3_ nanoparticles and loaded them with cisplatin for TD and SR of the drug at the cancerous cells [7,8]. CaCO_3_ nanoparticles encapsulated with cisplatin showed 70 wt% drug loading capacity and cargo releasing rates of 0.18%, 0.98%, 12.0%, 13.6%, and 16.4% at pH values 8, 7, 6, 5, and 4, respectively, thus showing appreciable rates at acidic pH values below 7.0 due to slow dissolution of the CaCO_3_ nanoparticle carriers, and thereby supplying both the drug and calcium ions. 

**Figure 1:**
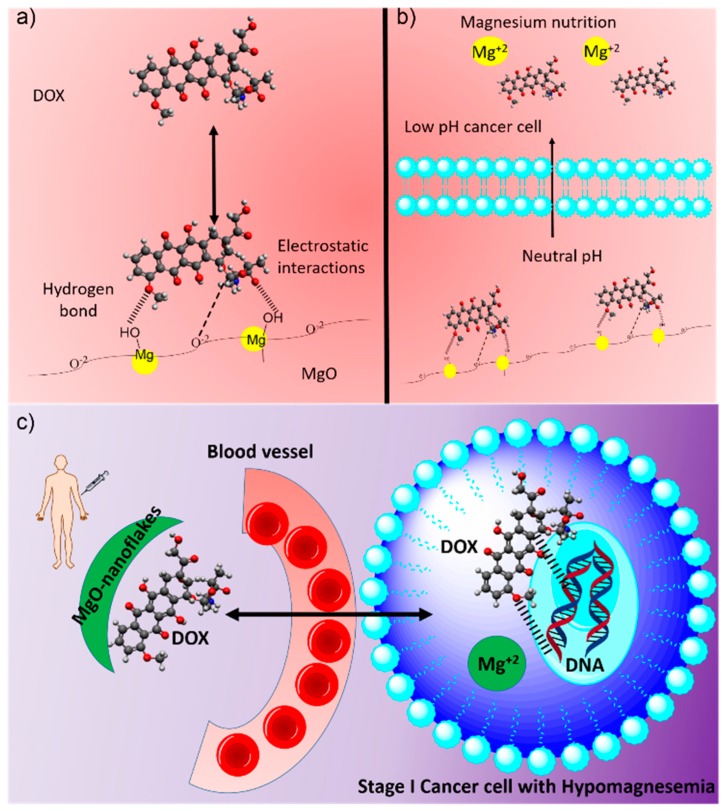
Schematic representation of MgO-DOX TDD system: Electrostatic interactions and hydrogen bonds facilitating binding of DOX on to MgO surface (**a**) and drug and Mg^2+^ slow release at slightly acidic conditions prevailing in cancer cells (**b**). After slow diffusion of MgO nano flakes from blood vessel to cancer cell, DOX molecules interact with mutated DNA and Mg^2+^ act as a nutrient to hypomagnesemia (**c**).

Inspired by Gan et al. [9], we advanced this study towards highly porous MgO nanoparticles exhibiting a flake-like morphology as a dual therapy agent. Counter to previously reported works [10], our MgO nanoparticles were synthesized [11,12] to produce pure materials void of potentially toxic metal cation contaminants [13]. The nanoparticles were characterized to confirm such, and their application as a pH-dependent TD drug carrier for the anticancer drug DOX was evaluated. For this study, DOX was utilized as a valuable anticancer drug that requires a localized release. DOX has been shown to affect multiple organs with damage to the heart and vascular system as the most common side effect [14]. A schematic illustrating the carrier design is shown in Figure 1. At the extracellular membrane of cancerous cells, the MgO carrier dissolves slowly, releasing both the DOX and magnesium ions. The latter acts an additional advantage to the properties of the drug carrier. Cancer cells have a propensity to absorb large amounts of magnesium ions, thereby depleting the magnesium in the surrounding biological environment. Such a decrease in magnesium levels in the blood leads to the development of an anemic condition known as hypomagnesemia [15,16,17]. This is also one of the most common side effects of chemotherapeutic drugs (e.g., cisplatin, cyclosporine, enzastaurin) [16]. The condition occurs when the blood magnesium concentration is reduced to below 1.8 mg/dL (0.70 mmol/L). A lack of magnesium ions can effect calcium-potassium regulation, cardiac and neural conduction, and adenosine triphosphate (ATP) metabolism. In addition, many enzymes that influence the metabolism of various biomolecules require magnesium ions as a source [17]. The drug carrier developed herein has the potential to act as a magnesium supplement to treat hypomagnesemia, as well as a cancer therapy agent.

## 2. Materials and Methods

Cetyltrimethylammonium chloride (CTAC) (98% purity), nitric acid (68% assay), barium chloride (99% assay), potassium chloride (98% assay), potassium monohydrogen orthophosphate (99% assay), sodium Chloride (99% assay), sodium bicarbonate (99% assay)_,_ magnesium chloride (99% assay), calcium chloride (98% assay), sodium sulfate (99% assay), sodium acetate (99% assay), potassium hydrogen phthalate (KHP) (99% assay), ethanol (99% purity), and acetic acid (99.5% assay) were purchased from Sigma-Aldrich (St. Louis, MO, USA). These chemicals were of analytical grade and used without further purification. Doxorubicin vial bottles containing 50 mg powder were purchased from Sri Lanka Pharmacy, Kandy, Sri Lanka. Bittern from Puttalam salt Ltd., Palavi, Puttalam, Sri Lanka (N 7.979826°, E 79.828478°) was used as a magnesium source and characterized by specific gravity measured using hydrometer according to the ASTM International standards (D1429) (ASTM International, 2017). Solutions of specific gravity >1.25 were used. For a 200.0 mL sample, 5.0 mL of 1.0 mol L^−1^ HNO_3_ was added and refluxed for 1 h to form a homogenous mixture. The sample was then treated with 0.5 mol L^−1^ barium chloride to remove sulphate ions. After precipitation, the suspension was filtered, and clear filtrate was collected. As the secondary magnesium source, dolomite obtained from marble quarry in Madawala Ulpatha, Sri Lanka (N 7.573308°, E 80.626237°) was used. 15.0 g of dolomite was mechanically crushed and ground in a sintered crucible. The powdered sample was sieved to obtain a portion of particles less than 150 µm in size. These particles were heated at 1000 °C for 2 h to produce calcined dolomite. Additional details are provided in the supporting information (SI).

MgO nanoparticles were synthesized as follows [18,19]: First, 100.0 mL of pre-treated bittern solution was taken to a 500 mL beaker and 1.0 mL of 1 mmol L^−1^ CTAC surfactant was added and stirred for 1 h. 2.0 g of calcined dolomite was then slowly added to the reaction mixture and thoroughly stirred for 12 h. The undissolved part was allowed to settle to the bottom of the beaker and the upper portion was composed of a gel. The gel was separated by filtration and heated in an oven at 100 °C for 24 h. The dry powder thus obtained was washed several times with distilled water and 5% ethanol. The product obtained was dried and calcined at 450 °C for 2 h in a furnace.

Fourier Transform Infrared (FT-IR) spectra of each product were recorded using a iS50 FT-IR instrument (Thermos scientific, Waltham, MA, USA) coupled with Attenuated Total Reflectance (ATR) technology. Samples were dried at 100 °C, and placed in a dry desiccator under a vacuum prior to analyses. The crystalline phases of the powdered products were analyzed by X-ray Diffractometry (XRD) using a D5000 Powder X-ray Diffractometer (Siemens, Munich, Germany) (Cu Kα radiation, λ = 0.1540562 nm, scan rate 1° min^−1^). The XRD patterns were analyzed with the aid of the ICDD PDF 2 database. The average crystallite sizes of magnesium oxide products were estimated by applying the Scherrer equation to the major XRD peaks.

Morphology and average size of particles and their aggregates were examined, after sputtering with gold particles, using an EVO LS 15 Scanning Electron Microscope (SEM, ZEISS, Germany) at an accelerating voltage of 20 kV. OXFORD analyzer (Oxford, England) coupled with the SEM was used to obtain energy-dispersive X-ray spectroscopic data (EDX). Particle size distribution was characterized by Laser Light Scattering based Particle Size Analysis (CILAS Particle Size Analyzer NANO DS, France). Nitrogen adsorption isotherms were measured at −196 °C on an ASAP 2010 volumetric analyzer (Micromeritics Inc., Norcross, GA, USA). Prior to adsorption measurements, all samples were out gassed under the vacuum at 110 °C for 2 h. Additional details are provided in the SI.

Preparation of simulated body fluid and cancer cellular fluid was carried out as follows. Simulated body fluid (SBF) is an acellular solution with composition and concentration similar to that of human plasma at 36.5 °C. The SBF was prepared by dissolving KCl, K_2_HPO_4_3H_2_O, NaCl, NaHCO_3_, MgCl_2_.6H_2_O, CaCl_2_, and Na_2_SO_4_ in distilled water and buffering at pH 7.4 with phosphate and HCl (1.0 M) at 36.5 °C as described in a previous study [20]. The pH 5.0 buffer was prepared by mixing 0.1 M sodium acetate (35.2 mL) and 0.1 M acetic acid (14.8 mL) and diluting to 100 mL to maintain acetate buffer in a SBF solution. The pH 3.0 buffer was prepared by mixing 0.1 M KHP (100 mL) with 0.1 M HCl (44.6 mL).

Drug loading study was performed using different concentrations of doxorubicin hydrochloride in the range 0.1–0.5 mg/mL. 1 mg/mL of MgO nanoparticles were used with each of the solution. A comprehensive study was carried out using 1 mg/mL solution of doxorubicin hydrochloride prepared from dry powder by dissolving 50 mg into 50 mL of double distilled water. 1.0 g of MgO nanoparticles were dispersed separately in 10 mL of each of these solutions. The mixture was first sonicated for 1 h, which was followed by overnight stirring at room temperature in the dark. The samples were centrifuged and washed several times to remove excess DOX. Centrifugation was performed at 5000 rpm until a colorless supernatant was observed leaving a purple residue at the bottom of the centrifuge tube. Aided by UV-Vis spectroscopy, drug loading capacity (DLC, wt.%) and drug loading efficiency (DLE, wt.%) were calculated by measuring concentrations of DOX in the solutions before and after loading. Additional details are provided in the SI.

In-vitro release studies were performed on prepared nanomaterials at 37 °C in three different media, i.e., (a) KHP buffer at pH 3.0 (b) acetate buffer at pH 5.0 (c) phosphate buffer at pH 7.4. Three portions of MgO nanoparticles (50 mg) were transferred into three dialysis membranes previously soaked overnight in 4.0 mL of buffer. After that, sealed membranes were introduced into three separate flasks containing 100.0 mL of corresponding buffer solution (pH 7.4, pH 5.0 and pH 3.0). Samples were shaken horizontally in a thermostat shaker at 37 °C and 100 rpm. At predetermined time intervals, a 4.0 mL sample of the medium was taken and replaced with the same amount of fresh buffer to maintain a constant volume. The release experiments were conducted in triplicate. The results presented are the average data of three repetitive measurements together with their standard deviations. DOX concentration in solutions was evaluated using UV-visible absorption spectroscopy by measuring absorbance at 253 nm since it is a narrow band with high molar absorption coefficients [21]. Furthermore, this band position that is determined by the π → π* transition of DOX is independent of the pH of the medium. However, the absorption band centered at 480 nm (n → π* transition) of DOX at pH 7.0 showed red shift as the pH of the medium was decreased (Figure S1). In order to convert absorbance data to concentrations, calibration curves were run with DOX in corresponding buffer solutions at the same pH values by measuring absorbance at 253 nm and the applicability of the Beer-Lambert law was verified (Figure S2). The cumulative release profile of DOX loaded to a MgO nanoparticle was obtained via the concentration correction (the amount of DOX in each aliquot was calculated to correct the overall cumulative releasing of DOX) of released DOX using Equation (1).

(1) $$c_{t}^{'}=c_{t}+\left(\frac{v}{V}\right)\overset{t-1}{\underset{0}{\sum }}c_{t}$$

where $c_{t}^{'}$ is the corrected concentration at time *t*, $c_{t}$ is the apparent concentration at time *t*, v is the volume of the aliquots taken, and V is the total volume of buffer.

## 3. Results and Discussion

### 3.1. Synthesis and Characterization of Magnesium Oxide Nanoflake

**Figure 2:**
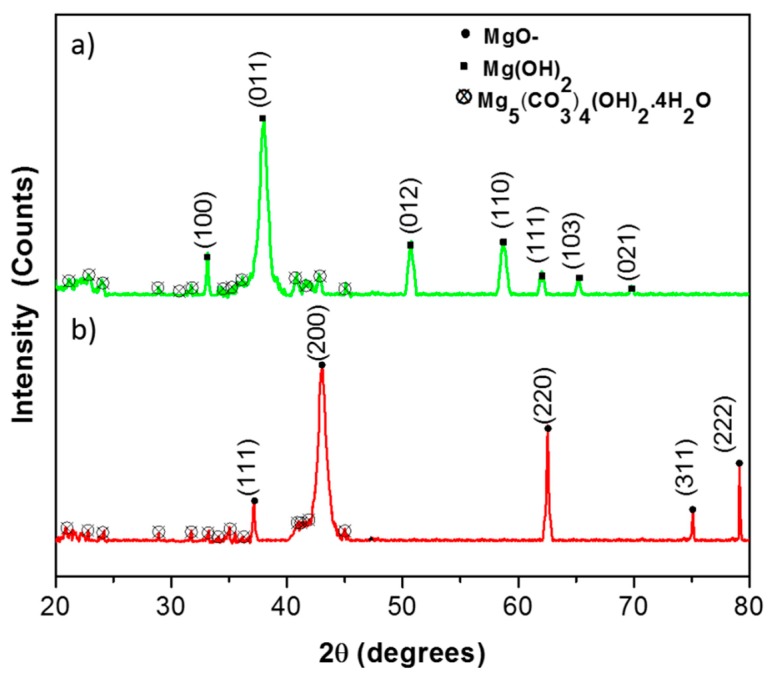
XRD pattern of before calcination (**a**) and after calcination of nanoparticles (**b**).

In this synthesis, magnesium ions react with hydroxyl ions and magnesium hydroxide (Mg(OH)_2_) nucleates within the cavities of soft templates formed by cetyltrimethylammonium ions. These nuclei grow to a certain size determined by the cavity volume. Other factors include interactions between the surfactants and Mg(OH)_2_ particles formed, and surface coverage of particles by surfactant molecules. Calcination of the Mg(OH)_2_ gel gives MgO nanoparticles. The X-ray diffractogrammes taken before and after calcination of the products are shown in Figure 2a,b, respectively.

The XRD pattern obtained before calcination consists of Mg(OH)_2_ peaks at 2θ values of 33.0°, 37.9°, 50.8°, 58.5°, 62.0°, 65.2°, and 69.8°, which correspond to diffractions from (100), (011), (012), (110), (111), (103), and (021) crystallography planes (Figure 2a). All of these peaks are assigned to brucite crystalline form of magnesium hydroxide-brucite [JCPDS card No. 82-2453] [18] The pattern shows a major XRD peak in the 2θ value of 37.9° that corresponds to diffraction from the (011) plane. Application of the Scherrer equation to this peak gives the average crystalline size to be 5 nm. 

The XRD peaks of MgO obtained after calcination of Mg(OH)_2_ appear at 2θ values of 37.1°, 43.1°, 62.4°, 75.3°, and 79.5° (Figure 2b), which correspond to diffractions from (111), (200), (220), (311), and (222) planes, respectively [JCPDS file: 75-1525] [18]. When the Scherrer equation is applied to the major XRD peak, at a value of 43.1°, a crystalline size of 20.0 nm is obtained. Increase of the average crystallite size of MgO may be due to the aggregation of particles when surfactant molecules are removed by combustion during heating. There is a minimum amount of hydro-magnesite (Mg_5_(CO_3_)_4_(OH)_2_.4H_2_O) present in both samples that has been formed due to adsorption of carbon dioxide from air.

**Figure 3:**
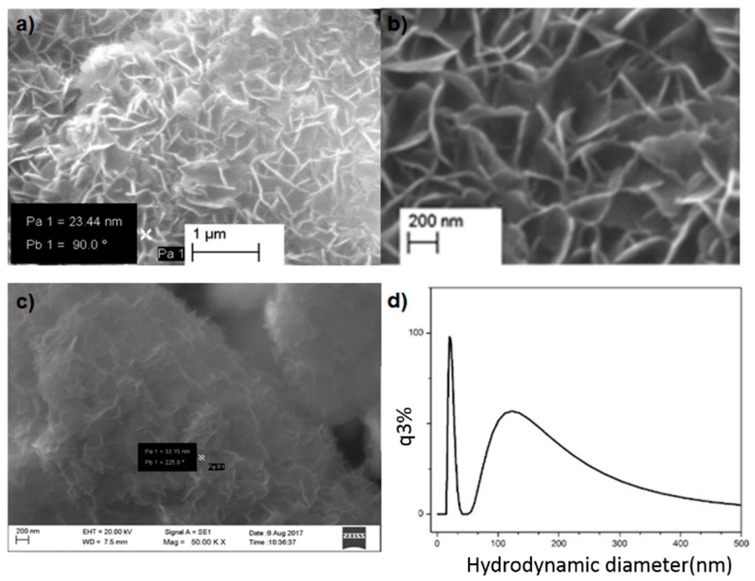
Surface morphology of prepared magnesium oxide nanoflakes 25,000× (**a**) and 50,000× (**b**) magnifications; (**c**) after drug loading and particle size distribution of synthesized nanoparticles (**d**).

Figure 3 shows SEM images at (a) 25,000× and (b) 50,000× magnifications. Images show a flake-like surface morphology. The flake-like morphology is obtained due to the self-assembly of CTAC ions facilitating the formation of Mg(OH)_2_ precursor particles. In these dimensions, the positively charged head of surfactant ions are stabilized by negatively charged hydroxyl ions, which are coordinated with magnesium ions. Therefore, in the presence of CTAC micelles, Mg(OH)_2_ forms a metal hydroxide coordination stabilized structure (Figure S3). The flakes have diameters in the range from 20 nm to 49 nm and widths from 80 nm to 400 nm. Particles tend to agglomerate to form a kind of porous structure, which enhances the surface area of the aggregated particle units.

Figure 3c highlights an SEM image of the sample obtained after drug loading. It is apparent that the flake-like morphology still remains, though the voids between the particles seem to have been filled with DOX. Particle size distribution measured from dynamic LASER light scattering based particle size analysis gives a distribution in the narrow range of 15 nm to 40 nm along the y-axis, and the range of 91 nm to 400 nm along the z-axis as shown by the two peaks in Figure 3d. Results obtained in independent measurements using two different techniques corroborate well, supporting a 2D morphology with similar nano-range dimensions.

The purity of the final product was assessed using EDX. The composition of the final product obtained was 98.6 wt% of MgO with 0.71 wt.% of sodium chloride and 0.69 wt% of silicon dioxide. Drug loaded nanoparticles were accounted for via EDX to confirm the elemental wt.% of carbon and nitrogen from DOX present in the final sample. Analysis shows a fair amount of carbon (13.75 wt%) which accounts for the total percentage of carbon from the drug bound to MgO nanoflaked particles. Nitrogen is due to the presence of amine groups in DOX molecules that accounts for 5.62 wt% by composition. In the final mixture, 18.45 wt% of magnesium is from the MgO particles themselves. A remaining 62.19 wt% is accounted for oxygen from both the drug and MgO.

### 3.2. Pore size Distribution of MgO Nanoflakes

**Figure 4:**
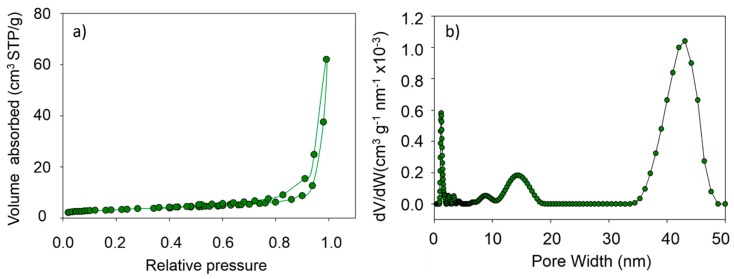
Nitrogen adsorption isotherms (**a**) and the corresponding pore size distribution curve (**b**) for MgO nanoflakes.

The N_2_ gas adsorption/desorption isotherm obtained for MgO nanoflakes is shown in Figure 4a. This type of adsorption isotherm corresponds to adsorption of gas molecules onto materials with pore sizes in the range of 1.5–100 nm. At higher pressures, the slope shows an increased uptake of adsorbate as pores become filled with an inflection point typically occurring near completion of the first monolayer. Figure 4b gives the pore size distribution derived from the N_2_ gas adsorption/desorption isotherm. Three bands between 8–10 nm, 10–18 nm, and 32–48 nm pore widths indicate the dimensions of the pores along three Cartesian coordinates. As such, it can be concluded that the pore sizes are in the nano-range of 50% porosity. Such high porosity of MgO nanoflake architecture is advantageous in accommodating large amounts of DOX molecules (*vide supra*).

### 3.3. FTIR Studies for Drug Loaded Nanoparticles

**Figure 5:**
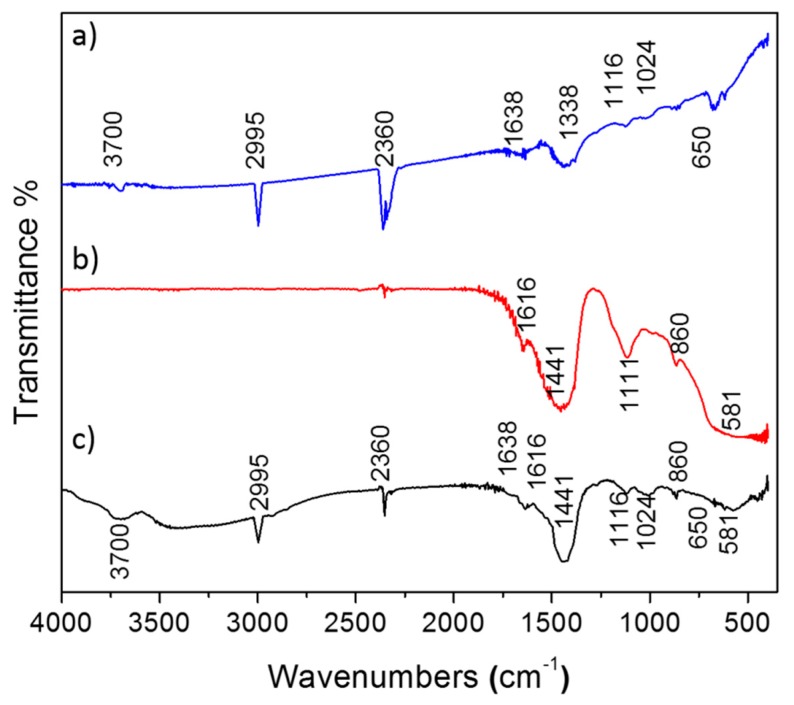
FT-IR spectrum of DOX (**a**) and MgO nano flakes (**b**) and MgO + DOX (**c**).

The FTIR spectrum of DOX, as depicted in Figure 5a, demonstrates several bands corresponding to stretching vibration of C–H at 2995 cm^−1^, O–H and N–H at 3700 cm^−1^, C–O–C at 1116 cm^−1^ and C–O at 1024 cm^−1^. Figure 5b represents the IR spectrum of MgO nanoparticles. The weak band appearing at 860 cm^−1^ indicates the presence of bending vibrations of the intercalated metal–oxide species [22]. The band at 1441 cm^−1^ is assigned to Mg–O bond vibration [23]. Broad absorptions observed at 581 cm^−1^ are generally regarded as a surface mode associated with lattice vibrations, which illustrates the fundamental lattice vibrations (phonon) of MgO nanoparticles due to the creation or annihilation of lattice vibrations.

The spectrum shown in Figure 5c is that of DOX-loaded MgO, which was recorded after drying the sample at 60 °C for 2 h in air. Some spectral changes such as appearance of new bands, change in intensity of previous bands. In addition, the broadening of some bands, together with shifts in band positions, are clearly apparent. The spectrum of parent DOX shows bands at 3700 cm^−1^ are due to N–H stretching vibrations, while 1638 cm^−1^ are due to N–H bend vibrations of the primary amine structure. However, in the case of DOX-loaded MgO, only a single broad band centered at 3440 cm^−1^ is observed and is due to the overlapping of N–H and O–H stretching vibrations. Comparison of the spectra suggest that there are strong interactions between DOX functional groups and hydroxyl groups of MgO nanoparticles possibly through hydrogen bonding. The appearance of characteristic bands of DOX in the encapsulated product confirm the presence of DOX molecules on the surface of MgO nanoparticles without any modification to DOX molecules. As such, it is possible that DOX molecules are bound to the surface of MgO nanoparticles via the electrostatic interaction of protonated NH_3_^+^ of DOX and O_2_^-^ sites on the surface of MgO nanoparticles as well as through hydrogen bonding between O-H and N-H groups of DOX and O-H groups of MgO nanoparticles.

### 3.4. Drug Loading Capacity (DLC) and Drug Loading Efficiency (DLE)

DLC and DLE are defined as shown in Equations (2) and (3) respectively.

(2) $$\mathrm{DLC}=\frac{\mathrm{amount}\;\mathrm{of}\;\mathrm{loaded}\;\mathrm{drug}}{\mathrm{amount}\;\mathrm{of}\;\mathrm{nanoparticles}}\times 100\%$$

(3) $$\mathrm{DLE}=\frac{\mathrm{amount}\;\mathrm{of}\;\mathrm{loaded}\;\mathrm{drug}}{\mathrm{amount}\;\mathrm{of}\;\mathrm{feeding}\;\mathrm{drug}}\times 100\%$$

Considerably high values of 90.20% and 92.44% are obtained for DLC and DLE, respectively, for the loading of DOX on MgO nanoflake architectures. Compared to the 70% DLC obtained for cisplatin on CaCO_3_ nanoparticles [8], this is a remarkable improvement in drug loading for our carriers. Such high values are due to the high porosity (50%) generated in the architecture of MgO nanoflakes, providing a space for DOX molecules to reside while keeping them in place through noncovalent interactions.

### 3.5. Drug Releasing Studies

**Figure 6:**
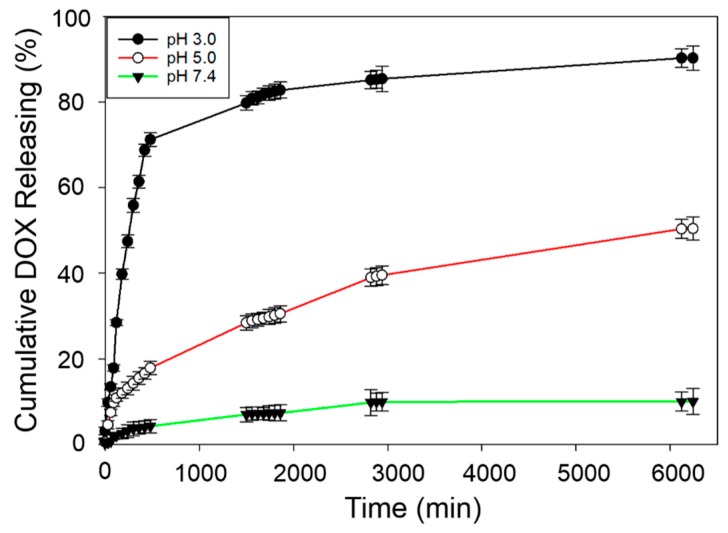
pH dependent drug release of DOX-entrapped MgO nanoflakes.

Kinetic analysis of drug release was performed for a 104 hour period by measuring absorbance values at 253 nm and converting them to concentration, *c_t_*, at time, *t*, and correcting to cumulative concentrations, $c_{t}^{'}$, using Equation (1). Additionally, the increase in ionic concentration of the buffer solutions to which DOX is released is verified by measuring electrical conductivity of the buffer solution as a function of time. An intital slow increase of electrical conductivity followed by rapid increase is obtained at each pH as more ions are released to the solution by slow dissolution. The rate of increase in conductivity is much higher at low pH—presumely due to the addition of magnesium ions releasing into the media—than that at a high pH (Figure S4). At pH 3.0, the drug is released rapidly within the first 8 hours as MgO dissolves readily at this pH, releasing 71.23% of the drug (Figure 6). The releasing kinetics at pH 5.0 and pH 7.4 show lower rates as MgO dissolves slowly at pH 5.0 and is not dissolved at pH 7.4, giving rise to 17.8% and 4.23% release within the first 8 h. The percentage of DOX released at the end of 104 h is 90.2, 50.5, and 7.4% at pH 3.0, 5.0, and 7.4 respectively. Data clearly show that the drug released at the physiological pH value of blood is insignificant during the transport of DOX in the carrier and slow release is obtained at the pH of the extracellular medium of the cancer cells. As such, MgO nanoflakes tend to entrap the drug in the cavities formed between the flakes. The flaky morphology affords strong binding of drug molecules for safe transport towards cancer cells where it can be released at a slow and steady rate alongside the magnesium ions. Therefore, this TDD system has dual advantages, as it could be used to treat cancer and hypomagnesemia simultaneously.

## 4. Conclusions

In this work, we report the synthesis of flake-shaped MgO nanoparticles with narrow particle size distribution. Nanoflakes agglomerate to form self-assembled structures with 50% porosity. Drugs such as DOX readily bind to the surfaces of these nanoflakes via hydrogen bonding and through electrostatic interactions. The inter-particle spaces of agglomerated nanoflakes can be filled up to 90.0% (loading capacity) with 92.4% drug loading efficiency. All materials synthesized were appropriately characterized by several independent analytical techniques with closely parallel results. The cumulative release kinetics of the drug were investigated at different pH values. Results corroborate the development of a novel material for loading DOX for pH dependent TDD to potentially treat both hypomagnesemia and cancer.

## Supplementary Materials

The following are available online at http://www.mdpi.com/2079-4991/9/2/208/s1. Figure S1: UV-VIS Spectra of standard solutions of Doxorubicin in different Buffer solutions at pH 3.0, 5.0 and 7.0. Figure S2: Calibration curves demonstrating Beer-Lambert Law for UV-visible spectroscopic determination of Doxorubicin at pH 7.4, 5.0 and 3.0 at 253 nm. Figure S3: Metal hydroxide coordination stabilized structure with CTAC micelle. Figure S4: Conductivity of buffer solutions as a function of log(time). Figure S5: XRD Pattern of Calcined Dolomite. Figure S6: (a) SEM image of Calcined Dolomite at 25000X magnification (b) EDX spectrum of Calcined Dolomite. Figure S7: Structure of Cetyltrimethylammonium chloride [CH_3_(CH_2_)_15_N(Cl)(CH_3_)_3_]. Table S1: Baumé Degree of Specific Gravity Conversion Chart. Table S2: Weight and atomic percentages obtained from EDX data.
