# Supplementary material for: Doxorubicin Loaded Magnesium Oxide Nanoflakes as pH Dependent Carriers for Simultaneous Treatment of Cancer and Hypomagnesemia
Source: Nanomaterials (Basel). 2019 Feb 6;9(2):208. doi: 10.3390/nano9020208 (PMC6409820; doi:10.3390/nano9020208)
Supplement: Supplementary file 1 [file nanomaterials-09-00208-s001.pdf]

Doxorubicin Loaded Magnesium Oxide Nanoflakes as pH Dependent Carriers for Simultaneous Treatment of Cancer and Hypomagnesemia

Tharindu A. Ranathunge [1,2,3,4], D.G.G.P. Karunaratne [2,3], R.M.G. Rajapakse [1,3,4,*] and Davita L. Watkins [4,*]

[1] Department of Chemistry, University of Peradeniya, 20400 Sri Lanka; garanath@go.olemiss.edu
[2] Department of Chemical and Processing Engineering, University of Peradeniya, 20400 Sri Lanka; dpkaru@eng.pdn.ac.lk
[3] Postgraduate Institute of Science, University of Peradeniya, 20400 Sri Lanka
[4] Department of Chemistry and Biochemistry, The University of Mississippi, University, MS 38677, USA
***** Correspondence: rmgr@pdn.ac.lk (R.M.G.R.); dwatkins@olemiss.edu (D.L.W.)

Table of Contents

ASTM Test method for Hydrometric Analysis of Brine: ASTM standard D1429 2

Unit conversion chart for specific gravity 2

UV-Visible Spectra of standard solutions of Doxorubicin in Buffer solutions at different pH values 3

Calibration Plots for Absorbance verses Concentration of Doxorubicin at (a) pH 7.4, (b) pH 5.0 and (c) pH 3.0 4

Structure of Cetyltrimethylammonium chloride [$CH_3(CH_2)_{15}N(Cl)(CH_3)_3$] 4

Conductivity of the Buffer Solutions 5

XRD Pattern of Calcined Dolomite 5

Surface Morphology and EDX Spectrum of Calcined Dolomite 6

EDX data: weight percentages and atomic percentages of 6

Debye-Scherer Formula 6

Structure of Cetyltrimethylammonium chloride [$CH_3(CH_2)_{15}N(Cl)(CH_3)_3$] 6

ASTM Test Method Using Hydrometer

- Summary of Test Method

Hydrometer is a weighted bulb with a graduated stem. It is used to determine specific gravity of liquids and is determined by the depth to which the hydrometer sinks in the liquid. The stem is graduated to directly read specific gravity of the liquid. Any oil present in the sample will interfere with the determination; therefore, only freshly filtered samples should be used.

- Apparatus

Hydrometer—A set of glass hydrometers (equipped with built-in thermometers) covering the range of specific gravities are used in water and brine analyses. They are graduated to read a minimum detection limit of 0.002. Hydrometer cylinder is made of clear glass, or plastic. For convenience in pouring, the cylinder may have a lip on its' rim. The inside diameter of the cylinder should be at least 25 mm greater than the outside diameter. The height of the cylinder is such that the hydrometer floats in the liquid sample with at least 25-mm clearance between the bottom of the hydrometer and the bottom of the cylinder.

- Procedure

The cylinder of the hydrometer is filled with the sample and the hydrometer together with the sample is freely floated in the liquid. It is then allowed to remain in the sample for 5 min until the thermometer to establish equilibrium reading. Both the specific gravity and the temperature are then directly read from the hydrometer stem and the attached thermometer.

- Correction of Specific Gravity to 60 °F (15.56 °C)

The specific gravity obtained at any other temperature above 60 °F may be corrected to 60 °F by adding to the value a correction factor obtained by multiplying the temperature difference in °F by 0.0002 for each degree above 60 °F as illustrated below.
Eg: Specific gravity of 1.1225 at 79 °F is corrected to 60 °F as follows.

$$\text{Correction} = (79 - 60) \times 0.0002 = 0.0038$$

$$\text{Specific gravity at } 60°F = 1.1225 + 0.0038 = 1.1263.$$

Unit conversion chart for specific gravity

Table S1. Baumé Degree of Specific Gravity Conversion Chart.

Degree Baumé	Specific Gravity		Degree Baumé	Specific Gravity	
	Heavier than Water	Lighter than Water		Heavier than Water	Lighter than Water
0	1		31	1.272	0.87
1	1.007		32	1.283	0.864
2	1.014		33	1.295	0.859
3	1.021		34	1.306	0.854
5	1.036		35	1.318	0.849
6	1.043		36	1.33	0.843
7	1.051		37	1.343	0.838
8	1.058		38	1.355	0.833
9	1.066		39	1.368	0.828

10	1.074	1	40	1.381	0.824
11	1.082	0.993	41	1.394	0.819
12	1.09	0.986	42	1.408	0.814
13	1.099	0.979	43	1.422	0.809
14	1.107	0.972	44	1.436	0.805
15	1.115	0.966	45	1.45	0.8
16	1.124	0.959	46	1.465	0.796
17	1.133	0.952	47	1.48	0.791
18	1.142	0.946	48	1.495	0.787
19	1.151	0.94	49	1.51	0.782
20	1.16	0.933	50	1.526	0.778
21	1.169	0.927	51	1.542	0.773
22	1.179	0.921	52	1.559	0.769
23	1.189	0.915	53	1.576	0.765
24	1.198	0.909	54	1.593	0.761
25	1.208	0.903	55	1.611	0.757
26	1.219	0.897	56	1.629	0.753
27	1.229	0.892	57	1.648	0.749
28	1.239	0.886	58	1.667	0.745
29	1.25	0.881	59	1.686	0.741
30	1.261	0.875	60	1.706	0.737

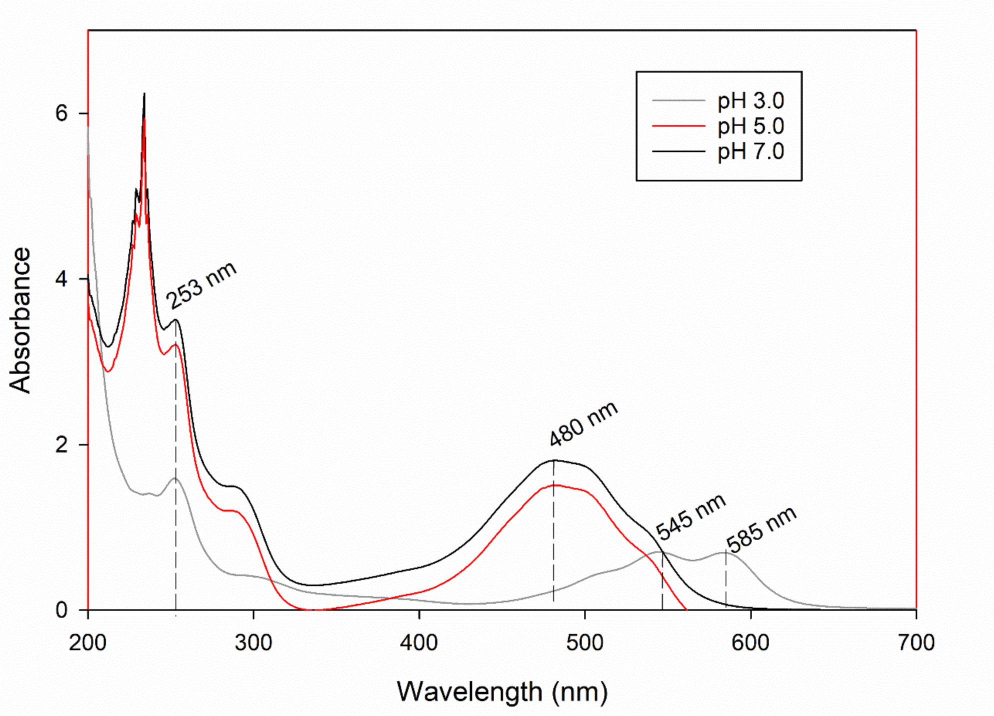

Figure S1. UV-VIS Spectra of standard solutions of Doxorubicin in different Buffer solutions at pH 3.0, 5.0 and 7.0.

UV-Visible absorption band positions of Doxorubicin (DOX) depends on pH of the medium. Figure SI-1 illustrates UV-visible absorption spectra of DOX in Buffered solutions of pH 3.0, 5.0 and 7.0, respectively. The absorption band centred at 253 nm which is due to $\pi \rightarrow \pi^*$ transition remains unchanged with the pH of the medium. However, its $n \rightarrow \pi^*$ transition shows red shift as the pH of the

medium is decreased. The n → π* transition of DOX appears at 480 nm at pH 7.0 and it remains so at pH 5.0. However, it shifts to 545 nm when the pH of the medium is 3.0 with an additional band centred at 585 nm. The absorption around 480 nm gives rise to red colour to the solution at pH 7.0 and 5.0 though the red shifting of this absorption is responsible for the purple colour at pH 3.0. Since the absorption band centred at 253 nm is sharp with high molar absorption coefficient it was used in the UV-visible spectroscopic determination of DOX in standard solutions as well as in the Buffer solutions to which DOX was released from MgO-DOX in dialysis bags in the DOX release kinetic study. Concentrations of the DOX was obtained from calibration plots which are given below. Excellent correlation coefficients (R^2) of 0.9986 and 0.9995 were obtained at pH 7.4 and 5.0 though it was 0.974 at pH 3.0.

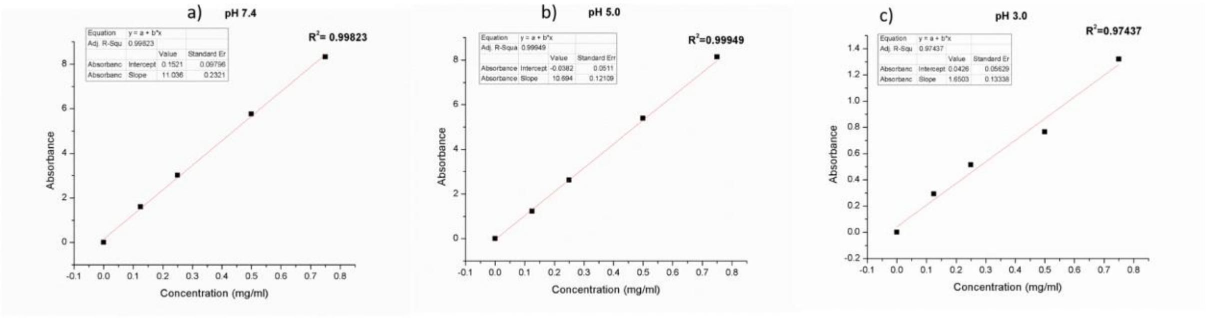

Figure S2. Calibration curves demonstrating Beer-Lambert Law for UV-visible spectroscopic determination of Doxorubicin at pH 7.4, 5.0 and 3.0 at 253 nm.

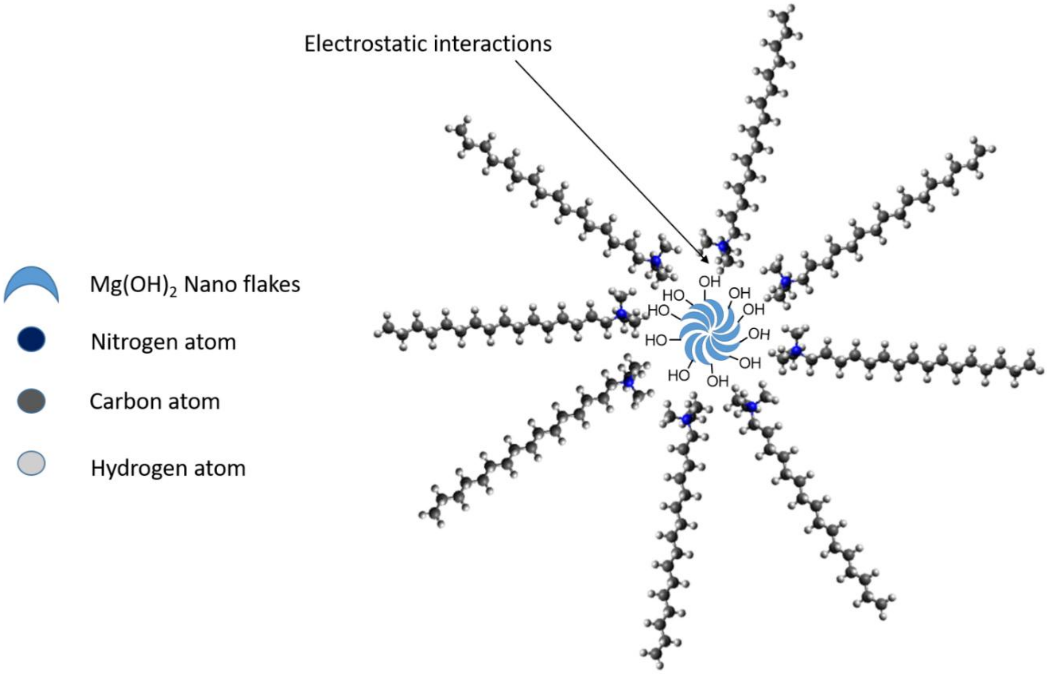

Figure S3. Metal hydroxide coordination stabilized structure with CTAC micelle.

Variation of Electrical Conductivity of buffer solutions as a function of log(time) for DOX release kinetic study from MgO-DOX solutions containing dialysis tubes immersed in buffer solutions of pH 3.0, 5.0 and 7.4.

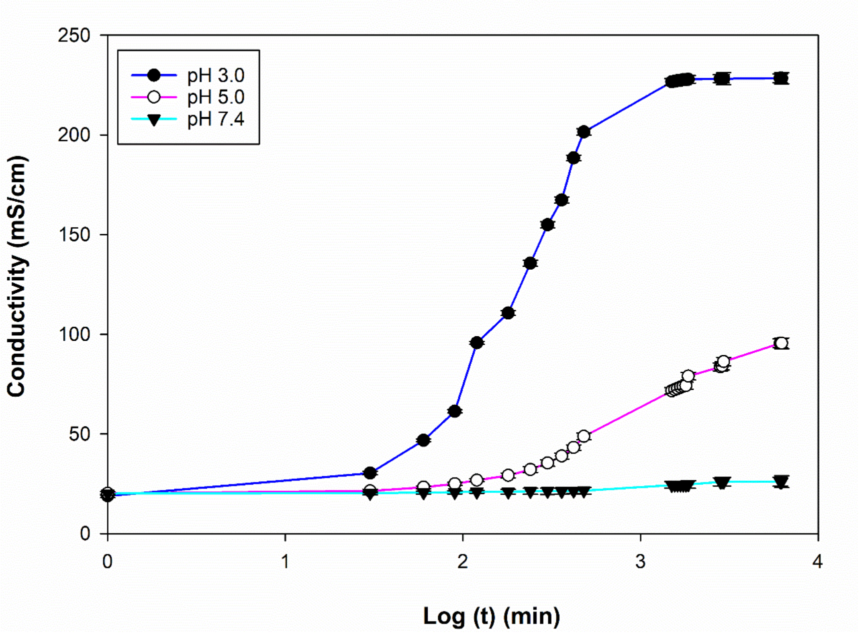

Figure S4. Conductivity of buffer solutions as a function of log(time).

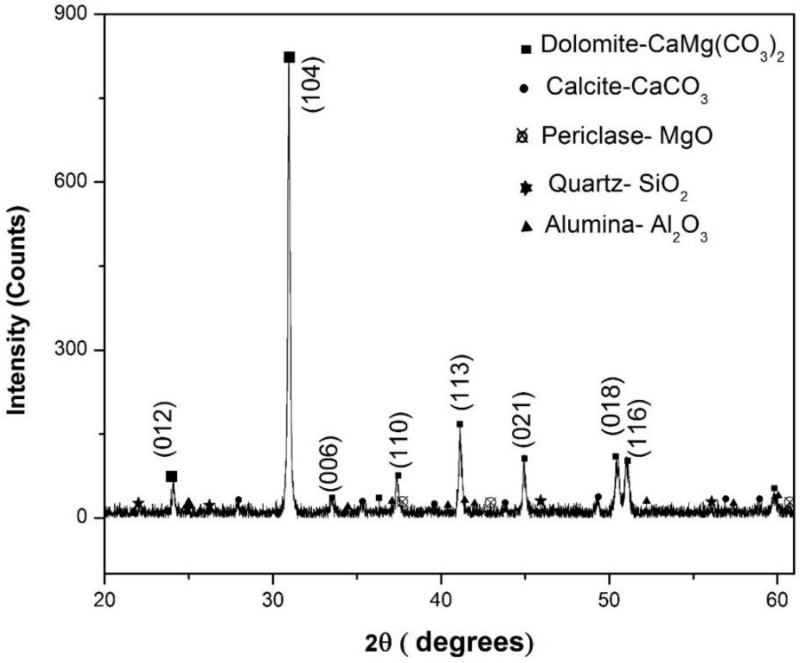

Figure S5. XRD Pattern of Calcined Dolomite.

Diffraction data identified in Figure S2, The major peak at a 2θ value of 30.9° indicates the presence of dolomite as the prominent mineral. The diffraction peaks observed at 2θ value of 24.7°, 30.9°, 33.4°, 37.3°, 42.1°, 45°, 50.5° and 51° represent the diffractions from Basal planes of (012), (104), (006), (110), (113),

(021), (018) and (116), respectively, of Dolomite [JCPDS 36-0426]. In addition to above dolomite, a minute amount of calcite, periclase, silica, and alumina particles are also present in the sample.

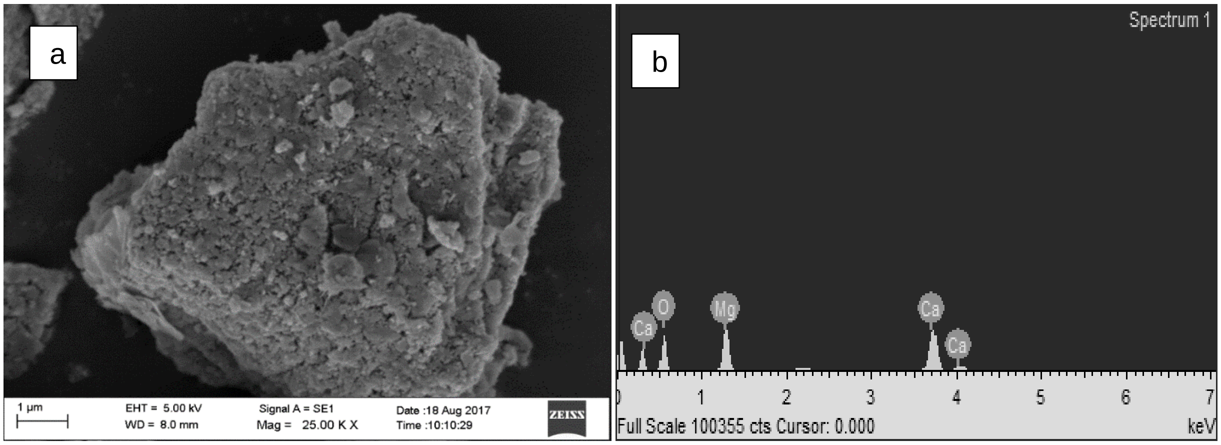

Figure S6. (**a**) SEM image of Calcined Dolomite at 25000X magnification (**b**) EDX spectrum of Calcined Dolomite.

Table S2. Weight and atomic percentages obtained from EDX data.

Element	Weight	Atomic%
O	67.60	80.14
Mg	14.66	11.44
Si	0.14	0.10
Ca	17.59	8.32
Total	100.00	100.00

Scherrer Equation

Peak broadening of the XRD reflection can be used to calculate the mean crystallite size (D) using the Scherrer equation, which is given in eq.(equation S1)

$$D = \frac{0:89\lambda}{\beta\cos\theta} \qquad \text{(equation S1)}$$

where λ is the X-ray wavelength of the Cu Kα radiation (1.54 Å), β is the full width at half maximum of the major XRD peak and θ is the angle of diffraction. Application of the Scherrer equation to the major XRD peak at 2θ value at 37.9° gives the average crystallite size of $Mg(OH)_2$ to be 5 nm.

Application of the Scherrer equation the major XRD peak at a 2θ value of 43.1° gives average crystallite size of synthesized MgO to be 20.0 nm. The increase of the average crystallite size of MgO may be due to aggregation of particles when surfactant molecules are removed by combustion during heating.

Structure of CTAC

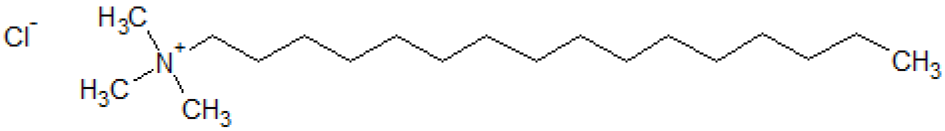

Figure S7. Structure of Cetyltrimethylammonium chloride [$CH_3(CH_2)_{15}N(Cl)(CH_3)_3$].
